# Adaptation of Carbon Source Utilization Patterns of *Geobacter metallireducens* During Sessile Growth

**DOI:** 10.3389/fmicb.2020.01271

**Published:** 2020-06-23

**Authors:** Sviatlana Marozava, Juliane Merl-Pham, Hubert Müller, Rainer U. Meckenstock

**Affiliations:** ^1^Institute of Groundwater Ecology, Helmholtz Zentrum München, Neuherberg, Germany; ^2^Research Unit Protein Science, Helmholtz Zentrum München, Neuherberg, Germany; ^3^Environmental Microbiology and Biotechnology, University of Duisburg-Essen, Essen, Germany

**Keywords:** microbial physiology, *Geobacter metallireducens*, label-free proteomics, sessile growth, carbon limitation, sediment columns

## Abstract

There are two main strategies known how microorganisms regulate substrate utilization: specialization on one preferred substrate at high concentrations in batch cultures or simultaneous utilization of many substrates at low concentrations in chemostats. However, it remains unclear how microorganisms utilize substrates at low concentrations in the subsurface: do they focus on a single substrate and exhibit catabolite repression or do they de-repress regulation of all catabolic pathways? Here, we investigated the readiness of *Geobacter metallireducens* to degrade organic substrates under sessile growth in sediment columns in the presence of a mixed community as a model for aquifers. Three parallel columns were filled with sand and flushed with anoxic medium at a constant inflow (18 ml h^−1^) of the substrate benzoate (1 mM) with non-limiting nitrate concentrations (30 mM) as electron acceptor. Columns were inoculated with the anaerobic benzoate degrader *G. metallireducens*. Microbial degradation produced concentration gradients of benzoate toward the column outlet. Metagenomics and label-free metaproteomics were used to detect and quantify the protein expression of *G. metallireducens*. Bulk benzoate concentrations below 0.2 mM led to increased abundance of catabolic proteins involved in utilization of fermentation products and aromatic compounds including the complete upregulation of the toluene-degrading pathway although toluene was not added to the medium. We propose that under sessile conditions and low substrate concentrations *G. metallireducens* expresses a specific set of catabolic pathways for preferred substrates, even when these substrates are not present.

## Introduction

The classical textbook knowledge of microbial growth states that microorganisms are only utilizing one substrate at a time while the consumption of other substrates is attenuated by catabolite repression leading to diauxic growth (Monod, 1942). However, there is strong evidence that such observations are mainly a laboratory effect from artificially high substrate concentrations used for cultivation (Silver and Mateles, 1969; Ihssen and Egli, 2005). Substrate concentrations in the environment are several orders of magnitude lower than what is usually applied in batch cultures (Egli, 2010). Simulating environmental conditions in chemostats revealed that gene regulation is very different from batch cultures growing at high substrate concentrations (Wick and Egli, 2004). In fact, even organisms that are adapted to rich media such as *Escherichia coli*, express almost all of their catabolic degradation pathways if grown under carbon limitation with glucose as the only electron and carbon source (Franchini and Egli, 2006).

In previous studies with *Geobacter metallireducens* as a model organism, we investigated whether this regulation patterns also apply to environmentally relevant biodegraders (Marozava et al., 2014a,b). *G. metallireducens* is a metal-reducing bacterium that is found in anoxic sediments contaminated with aromatic hydrocarbons (Lovley et al., 1993; Lovley, 1997). The physiology of *Geobacter* species has been widely investigated in batch (Merkley et al., 2015), chemostats (Yang et al., 2010), retentostats (Lin et al., 2009; Marozava et al., 2014b), as well as sediment incubations (Barlett et al., 2012), and subsurface environments (Wilkins et al., 2009, 2013).

Our experiments with extremely slow growth rates of 0.002 h^−1^ in retentostats revealed that *G. metallireducens* expressed many pathways for utilizing alternative carbon substrates not present in the cultivation medium (Marozava et al., 2014b). Similar results were found for the perchlorethylene biodegrader *Desulfitobacterium hafniense* Y51 (Marozava et al., 2018b) as well as for the nitrate-reducing monocyclic aromatics degrader *Aromatolium aromaticum* EbN1 (Trautwein et al., 2012) when they were cultivated in chemostats at nutrient-limiting conditions. This is presumably advantageous for bacteria in their natural habitats because they can immediately utilize carbon sources whenever they are available (Egli, 2010).

However, it remained unclear if the physiology of microorganisms in natural sediments can be fully simulated by chemostat or retentostat experiments. The difference to environmental conditions is the presence of high bacterial biomass and planktonic growth in chemostats, while natural conditions are characterized by very low biomass and mostly sessile life style in the presence of a mixed community. The main question remains if microorganisms in the environment utilize one substrate at a time or if they accommodate a de-repressed physiological state where all substrates are utilized simultaneously.

Hence, the aim of the current study was to elucidate how microorganisms utilize carbon sources in the environment. As an example of a typical microorganism found in groundwater, we cultivated *G. metallireducens* under conditions simulating sandy aquifers: in sediment columns, in the presence of a mixed community, and under steady flow of water containing the carbon substrate with subsequent development of concentration gradients along the sediment columns. In order to provide carbon-limiting conditions, an excess of electron acceptor (30 mM nitrate) was applied and other microorganisms were allowed to colonize columns. *G. metallireducens* is known to be a good competitor in microbial communities under acetate limitation (Li et al., 2020) and we expected similar scenario for benzoate limitation. The results were compared to our previously conducted studies where *G. metallireducens* was cultivated in batch and retentostats in the presence of excessive electron acceptor [50 mM Fe(III)citrate] (Marozava et al., 2014a,b). The physiology of *G. metallireducens* was investigated via proteomics to find out which catabolic pathways are expressed by such organisms in sediments.

## Materials and Methods

### Culture and Medium Preparations

*G. metallireducens* (strain GS-15/ATCC 53774/DSM 7210) was purchased from the Deutsche Sammlung von Mikroorganismen und Zellkulturen GmbH (DSMZ), Germany. Microorganisms were cultivated under anoxic conditions in a modified freshwater medium (Lovley and Phillips, 1986) consisting of NaHCO_3_: 2.5 g L^−1^; NH_4_Cl: 1.5 g L^−1^; NaH_2_PO_4_: 0.60 g L^−1^; KCl: 0.10 g L^−1^; Na-benzoate: 1 mM; 0.5 mM Fe(III)-citrate; pH 6.8; 30 mM NaNO_3_ as electron acceptor with DSMZ 1 mL L^−1^ trace element solution SL-10 (DSMZ Medium 320 for *Clostridium cellulovorans*) and 0.5 mL L^−1^ DSMZ 7 vitamins solution (DSMZ medium 503, anoxic freshwater medium) and kept under an anoxic atmosphere (N_2_:CO_2_; 90:10) (DSMZ). Fe(III)-citrate (0.5 mM) was used to provide optimal growth for *G. metallireducens* as it has been shown earlier that nitrate reduction of *G. metallireducens* is iron dependent and 0.5 mM of Fe(III)citrate increase the activities of nitrate reductases significantly (Senko and Stolz, 2001). Besides, *G. metallireducens* does not use citrate as an additional carbon source (Senko and Stolz, 2001) but other microorganisms in the community could use it. Inocula were pre-cultivated in 100 ml glass bottles. Medium used for column experiments was prepared in 20 L bottles and was constantly flushed with a mixture of N_2_:CO_2_ (90:10). Medium for batch cultivation (which was used for column inoculation and as a reference for proteomic analysis) was dispensed in 200 ml sterile bottles (triplicates), flushed with a mixture of N_2_:CO_2_ (90:10) and sealed with butyl stoppers.

### Cultivation of *G. metallireducens* in Columns

The columns were built by the mechanics workshop at Helmholtz Zentrum München. Columns were made from plexiglass, with a length of 50 cm and an inner diameter of 5 cm. Columns were sterilized with 70% ethanol for 1 h and filled with either natural sediment or coarse quartz sand with homogenous grain size of ~1 mm. Hereafter, quartz sand in Column 2 and 3 will be referred as sediment as it was used as a model for the groundwater sediment. Natural sediment was collected from a gravel pit in Bruckmühl near Munich, Germany and sieved with exclusion of grain sizes above 1 mm. The natural sediment and quartz sand were thoroughly washed five times in demineralized water, sterilized three times at 120°C and then dried at 105°C for 1 day. Columns were packed with dry sediment in a clean bench. To ensure homogeneous packing and to prevent air bubbles entrapment, columns were placed into a sonicator bath and mild sonication was applied after each stepwise filling with the sediment. In order to remove any remaining air bubbles, columns were flushed with sterile ultrapure MilliQ® water with a total organic carbon content (<10 μg L^−1^) at 0.25 ml h^−1^ for 1 week. Water or medium were injected at the columns bottom via a peristaltic pump. Due to the experimental set up, conditions were not sterile which also led to the colonization of the columns with other microorganisms.

In order to monitor stability of anoxic conditions for cultivation of *G. metallireducens*, oxygen sensor spots (Rhizosphere Research Products bv, Wageningen, the Netherlands) were installed inside the columns with a spatial resolution of 5 cm. Oxygen sensor spots were calibrated and installed according to the guidelines of the manufacturer. Oxygen was measured optically with optodes from the outside of the columns. Ports for pore water sampling with a diameter of 2.5 mm and a mean pore size of 0.15 μm (Rhizosphere Research Products bv, Wageningen, the Netherlands) were installed in the middle of each 5 cm section of the columns.

After conducting a tracer experiment and establishment of anoxic conditions, columns were equilibrated with sterile medium at 18 ml h^−1^ for 2 days. Then, ~140 ml of *Geobacter* culture, pre-grown with benzoate and nitrate were collected at the exponential phase with approximate density of 10^7^ cells ml^−1^ and was injected at the bottom of the columns with syringes. During injection, the medium was pumped through the columns in order to distribute bacteria along the whole length. After injection of bacteria, the flow of the medium through the columns was stopped for 1 day to allow bacteria to settle on the sediment. Afterwards, the flow was continued at a rate of 18 ml h^−1^. At the end of cultivation, the medium flow was stopped and the sediment cores of the columns were cut into 10 fractions of 5 cm. The sediment within each fraction was carefully homogenized with a sterile spatula. 0.5 ml of the sediment (in duplicates) was collected for flow cytometry; 40 g of the sediment (in duplicates) was collected for proteomic and metagenomic analysis.

### Tracer Experiment

A bromide tracer experiment was conducted to check the packing and performance of the columns prior to inoculation with bacteria. The columns were flushed with ultrapure water (MilliQ) at 0.3 ml min ^−1^ and 3 ml of a Br^−^ tracer solution (KBr, Sigma Aldrich, St. Louis, Missouri, USA) (70 mg L^−1^) was injected with a syringe through a septum. The outflowing water was collected in 30 min fractions using a fraction collector. The bromide tracer experiment was performed for Columns 1 and 2 and confirmed that the sediment was packed homogenously (Figure S1).

### Analytical Measurements

Benzoate was measured by HPLC (Shimadzu, Japan) using a PFP Kinetex column (75 x 4.6 mm) (Phenomenex Inc., USA). Elution was isocratic with 1% acetic acid in MilliQ water (solvent A) and 1% acetic acid in methanol (solvent B) (50:50, v:v) at a flow rate of 0.7 ml min^−1^ (UV detection at 236 nm). Column temperature was set to 30°C.

Bromide was analyzed by ion chromatography on a DX-100 (Dionex, Germering, Germany) as described in Stoewer et al. (2015).

### Flow Cytometry

Duplicate 0.5 ml aliquots of each sediment fraction were fixed with 2.5% glutardialdehyde and stored at 4°C until further analysis. Sample preparation and flow cytometer analysis were done as described in Anneser et al. (2010). Briefly, glutardialdehyde was removed from the sediment via centrifugation for 10 min at 17,900 g. Fixed cells were detached from the sediment via shaking in a swing mill (Retsch, MM 200) for 3 min at 20 Hz in the presence of 1.5 ml of phosphate-buffered saline (PBS: 3 mM sodium phosphate, 150 mM sodium chloride, 1.05 mM potassium phosphate, pH = 7.2). Then, cells were separated from the sediment particles via gradient centrifugation at 12,900 g for 1 h at 4°C in the presence of a Nycodenz gradient with a density of 1.3 g ml^−1^ (Nycomed Pharma AS, Oslo, Norway) in an ultracentrifuge (Optima XE-90, Beckman Coulter). After centrifugation, the upper layer of the supernatant (1.5–2 ml) was harvested. Collected cells were diluted 10 times with PBS buffer, stained with SYBR-Green I (Molecular Probes, Invitrogen, Karlsruhe, Germany) with a 10,000-fold dilution of a stock solution for 15 min in the dark and then subjected to flow cytometry (Cytomics FC 500 CXP, Beckman Coulter, Krefeld, Germany) together with TruCount beads (TruCount tubes, Becton Dickinson, Heidelberg, Germany) as internal standard. The following settings were used for flow cytometry: forward scatter (FS) 398 mV, side scatter (SS) 972 mV. The cell numbers were calculated according to the formula described in Nebe-von-Caron et al. (2000).

### Protein Extraction

Sediment collected for proteomics analysis was immediately put at −80°C and stored until further analysis. Proteins were extracted according to a protocol modified after (Taylor and Williams, 2010) and (Keiblinger et al., 2012). Briefly, sediment samples were thawed at room temperature. PBS buffer (30 ml per 10 g of sediment) was added to sediment in 50 ml falcon tubes and tubes were shaken in a swing mill for 3 min at 20 Hz. The supernatant containing the cells was taken immediately and centrifuged at 13,000 g, 4°C, 10 min (Beckman Coulter, Krefeld, Germany). The pellet was homogenized in a lysis buffer [0.05 M disodium ethylenediamine tetraacetate•2H_2_O (EDTA), 0.9 M sucrose, 10 mM dithothreitol (DTT), 1% sodium dodecyl sulfate (SDS)] and sonicated twice for 2 min (Ultrasound processor UP50H, Hielscher, Germany; 0.5 s per pulse, 60 % duty) with sample cooling on ice for 1 min between the rounds. One milliliter of phenol was added per each extraction and samples were shaken for 1 h at RT, 500 rpm. Extractions were centrifuged at 18,000 g, 4°C, 12 min (Beckman Coulter, Krefeld, Germany). Then, the upper phase was transferred into 5 x volume ice-cold 0.1 M ammonium acetate in methanol and kept overnight at −20°C. On the next day, the samples were centrifuged at 13,000 g, 0°C, 10 min. Pellets were homogenized, washed twice in 80% acetone and air-dried. Further, protein pellets were dissolved in urea buffer (9 M urea, 2 M thiourea, 4% CHAPS, 65 mM DTT).

Cells from batch cultures were harvested during exponential phase and extracted according to Marozava et al. (2014a).

Protein concentration was determined using the Bradford protein assay (Bio-Rad, Munich, Germany) with bovine serum albumin as the standard (Bradford, 1976).

### Proteomic Sample Preparation and LC-MS/MS -Measurement and -Analysis

Each 10 μg of every column fraction and of the batch sample were digested using a modified filter-aided sample preparation procedure (Wiśniewski et al., 2009; Grosche et al., 2016). Briefly, proteins were reduced and alkylated using dithiothreitol and iodoacetamide, and diluted to 4 M urea prior to centrifugation on a 30 kDa filter device (PALL). After several washing steps using 8 M urea and 50 mM ammonium bicarbonate, proteins were digested on the filter by 1 μg Lys-C for 4 h at room temperature and 2 μg trypsin over night at 37°C. Generated peptides were eluted by centrifugation, acidified with TFA and stored at −20°C.

Samples were measured on a LTQ OrbitrapXL mass spectrometer (Thermo Scientific) online coupled to an Ultimate 3000 nano-RSLC (Dionex) as described previously (Hauck et al., 2010). Tryptic peptides were automatically loaded on a C18 trap column [300 μm inner diameter (ID) × 5 mm, Acclaim PepMap100 C18, 5 μm, 100 Å, LC Packings] prior to C18 reversed phase chromatography on the analytical column (Acclaim PepMap C18, 50 μm ID x 250 mm, 2 μm, 100 Å, LC Packings) at 300 nl min^−1^ flow rate in a 140 min acetonitrile gradient from 4 to 30% in 0.1% formic acid. Profile precursor spectra from 300 to 1,500 m/z were recorded in the orbitrap with a maximum injection time of 500 ms. TOP10 fragment spectra of charges 2 to 7 were recorded in the linear ion trap with a maximum injection time of 100 ms, an isolation window of 2.0 m/z, a normalized collision energy of 35 and a dynamic exclusion of 60 s.

Generated raw files were analyzed using Progenesis QI for proteomics (version 2.0, Non-linear Dynamics, part of Waters) for label-free quantification as described (Hauck et al., 2010; Merl et al., 2012). After alignment and global normalization, features of charges 2–7 were used and all MS/MS spectra were exported as mgf file. Peptide search was performed using Mascot search engine (version 2.5.1) against the below described in house metagenome database (339,561 sequences, 98,128,740 residues) which was created via metagenome-sequencing of selected column fractions as described below. Search settings were: 10 ppm precursor tolerance, 0.6 Da fragment tolerance, one missed cleavage allowed, carbamidomethyl on cysteine as fixed modification, deamidation of glutamine and asparagine allowed as variable modification, as well as oxidation of methionine. Applying the percolator algorithm (Brosch et al., 2009) resulted in a peptide false discovery rate (FDR) of 0.39%. Search results were reimported in the Progenesis QI software. Proteins were quantified by summing up the abundances of all unique peptides per protein. Resulting protein abundances were used for calculation of fold-changes and statistics values. Further analysis was focused on the proteins of *G. metallireducens* and their function was derived from COG and KEGG databases (Data Sheet S1).

### Detection of Significantly Abundant Proteins

Normalized and log10 transformed protein abundances of *G. metallireducens* were used to identify differentially expressed proteins using a linear model for microarray data analysis (LIMMA) in Bioconductor (Smyth, 2005; Ritchie et al., 2015) where selected pair-wise comparisons were tested. A false discovery rate was estimated using the Benjamini–Hochberg method (Benjamini and Hochberg, 1995). Visualization of hierarchical clustering of z-score-normalized protein abundances (based on Euclidean distance) and conditions was done via creating heatmaps in Perseus.

### Assembly-Based Metagenomics

Since *G. metallireducens* was grown in the columns in the presence of a mixed community, metagenomic analysis was performed to assist in identification of *G. metallireducens* proteins. Although the genome of *G. metallireducens* is known, knowledge on the genomes of other bacteria improves the identification of proteins of the organism of interest.

For metagenomic analysis, DNA was extracted from sediments of different column fractions (30 samples in total) as described in Marozava et al. (2018a). The microbial diversity in the column fractions was determined by bacterial 16S rRNA gene-targeted terminal restriction fragment length polymorphism (TRFLP) fingerprinting (Marozava et al., 2018a). TRFLP fingerprints were used to identify fractions with the most bacterial diversity. Nine fractions with the highest diversity across all columns were selected as representatives for the microbial community. The fractions which were selected for metagenomic analysis are indicated in Figure S2A.

Assembly-based metagenomics was performed on DNA samples extracted from the sediments. Nine DNA samples were sent to GATC (Konstanz, Germany) for library preparation and 150-bps paired-end Illumina HiSeq sequencing. Raw reads were processed by trimming and quality filtering with bbduk (http://jgi.doe.gov/data-and-tools/bbtools/) and SICKLE version 1.21 (https://github.com/najoshi/sickle). Afterwards, reads were assembled and scaffolded with metaSPADES version 3.10.1 using default settings (Nurk et al., 2017). For scaffolds longer than 1 kb, genes were predicted with prodigal in the meta mode (-p meta) (Hyatt et al., 2010) and annotated using diamond blastp (Buchfink et al., 2015) against the Uniref100 database (Suzek et al., 2014). 16S rRNA genes were identified using CMsearch as part of the Infernal software package (Nawrocki et al., 2009; Brown et al., 2015). Raw sequencing reads were deposited in Bioproject ID PRJNA576198.

## Results

### Bacterial Growth and Degradation of Benzoate in Columns

*G. metallireducens* was inoculated to three sediment columns which led to establishing of gradients of benzoate concentrations in the 50 cm between inlet and outlet. The replicates differed slightly in the experimental setup in order to reproduce the principles of bacterial growth in the sediments rather than to produce three identical gradients.

**Figure 1:**
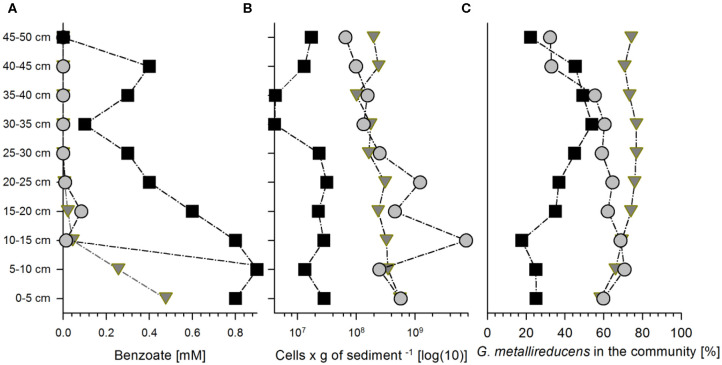
Benzoate concentrations and bacterial abundances in the three sediment columns at the end of the cultivation at 300, 528, and 552 h for columns 1 (squares), 2 (circles), and 3 (triangles), respectively. Concentrations of benzoate **(A)**, total bacterial cell counts **(B)**, and relative abundance of *Geobacter metallireducucens* in the communities according to the expressed percentage of normalized protein abundances of *G. metallireducens* from all the proteins detected **(C)** along the length of columns (for the distribution of the other community members please refer to the Figure S2).

Column 1 (packed with natural sediment) was harvested after 12.5 days of cultivation, while the other two columns (packed with quartz sand also named sediment hereafter) were harvested after 22 (Column 2) and 23 (Column 3) days of cultivation, respectively. Degradation of benzoate in Column 1 produced an almost linear gradient from 0.9 mM at the inlet to non-detectable concentrations at the outlet (Figure 1A). Columns 2 and 3 showed very steep gradients of benzoate along the first 10 cm (1–1.1 and 0.48–0.26 mM in column 2 and column 3, respectively) to very low concentrations in the following column fractions (ranging from 0.1 mM to non-detectable) (Figure 1A).

We added an excess of nitrate to ensure that the system is electron donor-limited at any spot in the columns. Hence, concentrations in the columns did not decline significantly, whereas 6 mM nitrate are enough to oxidize 1 mM of benzoate to CO_2_.

The total bacterial abundances decreased only slightly from the inlet region of 0–10 cm depth to the outlet region of the three columns at 40–50 cm depth. Bacterial abundances ranged from 4·10^6^ to 3·10^7^ cells per g of sediment (Column 1), 7·10^7^ to 7·10^9^ cells per g of sediment (Column 2), and 1·10^8^ to 6·10^8^ cells per g of sediment (Column 3) (Figure 1B). The columns were not maintained at strictly sterile conditions which led to colonization with other microorganisms. This produced diverse bacterial communities (Figure S2) where the average relative abundance of *Geobacter* proteins ranged from 22% to ~53% of the total community proteins in Column 1 and to 71 and 77% in Columns 2 and 3, respectively (Figure 1C, Figure S2).

### Protein Expression in the Sediment Columns

At the end of the cultivation, the proteins were extracted from the sediment including pore water from every 5 cm depth and analyzed by mass spectrometry. Since the percentage of sessile organisms in sediments vastly exceeds the planktonic ones in the pore water, mostly sessile bacteria were analyzed in the metagenomic and metaproteomic analysis. Metagenomic analysis was conducted to assist differentiating *G. metallireducens* polypeptides from the pool of proteins from other bacteria.

In total, proteins from 87 different bacterial taxa were identified via the metaproteomic approach where peptide search was done against an in house metagenomic database created via metagenomic analysis of the selected column fractions (Data Sheet S1). Several identified taxa are known benzoate degraders affiliated to the genera *Acinetobacter* (up to 30% of all bacteria in Column 1), *Cupriavidus* (up to 25% of all bacteria in Column 3), and *Comamonas* (up to 32% of all bacteria in Column 2) (Figure S2).

3,741 proteins were detected in the total proteome, 1,004 of which belonged to *G. metallireducens* (Data Sheet S1). The distribution of all proteins of *G. metallireducens* analyzed across the clusters of orthologous groups (COG) categories revealed that the highest represented categories were translation as well as amino acids transport and metabolism (53 and 46% of all detected proteins, respectively). The least expressed proteins were from the category cell motility (one protein detected out of 130 proteins predicted). The other categories ranged between 10 and 40% of the predicted proteins (Figure S3).

**Figure 2:**
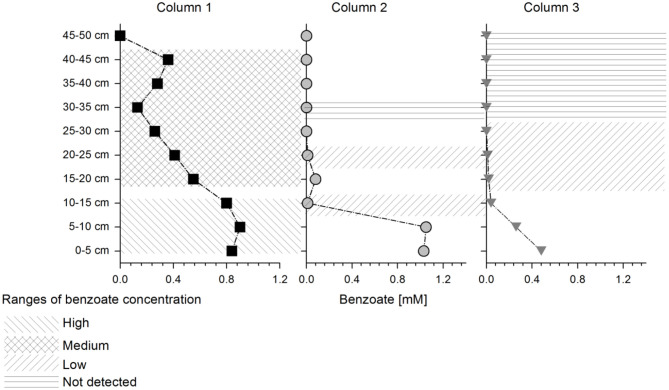
Schematic representation of selected depths for statistical analysis: Different shaded areas on the columns represent different benzoate concentration ranges which were used further for the pairwise comparisons (as indicated in the legend). The columns are represented with the following symbols: columns 1 (squares), 2 (circles), and 3 (triangles), respectively.

Non-parametric clustering of all proteins detected at each column depth showed that Column 1 (natural sediment) differed from Columns 2 and 3 (quartz sediment) and all columns showed different protein expression than the batch cultures, which were used as a reference (where *G. metallireducens* was also cultivated with benzoate and nitrate as electron donor and acceptor) (Figure S4). Three samples of Column 2 at depths 35–50 cm differed from the other samples at similar concentration range (Figure S4). These samples, as well as other samples where the concentration changed too fast leading to undefined conditions (fractions at depth 0–10 cm at Column 2 and 3; depth 45–50 cm at Column 1), were removed from further analysis. The columns and depths were sorted in four benzoate concentration ranges: high (~0.9 mM), medium (0.5–0.2 mM), low (0.01 mM) and below detection (benzoate detection limit of 0.02 mM) as indicated in Figure 2. Non-parametric clustering of the data from the selected depths showed a clear separation of expression profiles between high and medium benzoate concentration zones. Low and below detected areas clustered together (Figure S5).

**Figure 3:**
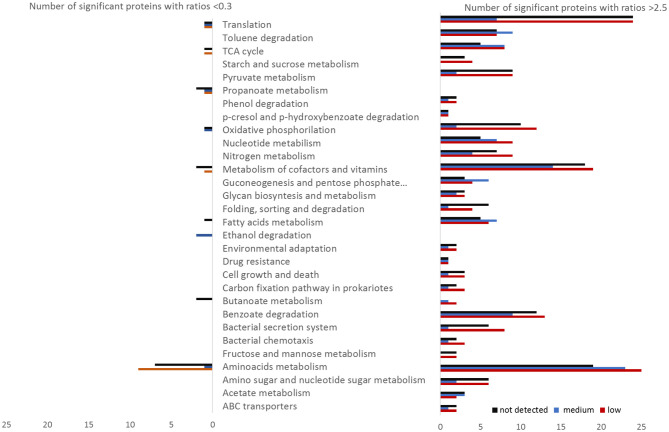
Number of *Geobacter* proteins with significantly different abundances (ratio above 2.5 or below 0.3) at low (red), medium (blue), or not detected (black) benzoate concentrations relative to the reference condition of high benzoate concentration in the column 1. The proteins were sorted by functional categories according to KEGG.

The proteomes of the different zones of the columns were compared to the high benzoate zone in Column 1 (~0.9 mM) (Figure S6). Pairwise comparison showed that most of the proteins were significantly downregulated in the high benzoate zones (~0.9 mM) relative to lower or no benzoate detected, clearly shown by the heatmap clustering of significant protein abundances (Figure S7). Proteins with increased abundances covered all parts of the metabolism, including catabolic pathways (Figure 3). The KEGG categories with the highest number of proteins with significantly increased abundances relative to high benzoate concentrations were translation, metabolism of cofactors and amino acids metabolism (Figure 3). However, the highest differentially abundant proteins were from catabolic pathways (91 proteins in total).

### Key Proteins of Carbon Metabolism

#### Acetate Metabolism

Most of the predicted proteins for acetate metabolism were detected in the three columns. Acetate kinase (Gmet_1034) showed increased abundances at the column areas with low benzoate concentrations, relative to high (Table 1). In retentostat experiments with *G. metallireducens* at low growth rates and low substrate concentrations (Marozava et al., 2014b), acetate kinase was also more abundant relative to batch conditions, suggesting that this enzyme is expressed in response to low substrate concentrations.

**Table 1:** List of differentially expressed catabolic proteins identified in Bioconductor (please refer to Material and methods) where the following pair-wise comparisons were performed between the column fractions with different benzoate concentrations: “medium” vs “high,” “low” vs “high,” and “below detection” vs “high.”

**Annotated Proteins**	**Averaged protein abundances relative to condition “high” (ratios)**		
	**Medium**	**Low**	**Not detected**
**Acetate metabolism**			
Q39WV0 Acetate kinase	***4.8***	***6.6***	***5.3***
**Benzoate degradation**			
Q39TP8 Metal-dependent hydrolase, putative	***8.3***	***5.5***	***4.6***
Q39TV9 putative benzoyl-CoA reductase electron transfer protein	***7.1***	***12.1***	***10.2***
Q39UP3 Benzoyl-CoA reductase, putative	***4.8***	***3.4***	***2.7***
Q39TX3 3-hydroxyacyl-CoA dehydrogenase	***4.3***	***3.1***	1.4
Q39TV7 6-oxocyclohex-1-ene-1-carbonyl-CoA hydrolase	***3.9***	***3.4***	***4.1***
Q39TP3 Electron transfer flavoprotein, alpha subunit	***3.6***	***2.8***	***3.1***
Q39TP2 Electron transfer flavoprotein, beta subunit	***3.1***	***2.8***	***2.8***
Q39QM3 Ferritin-like domain protein	***2.9***	1.1	0.8
Q39XP3 Sodium/solute symporter family protein	2.6	***4.5***	***5.4***
Q39TP4 6-hydroxycyclohex-1-ene-1-carbonyl-CoA dehydrogenase	***2.6***	***3.2***	1.5
Q39TP6 Lipoprotein, putative	***2.4***	***3.2***	***3.8***
Q39TZ1 Succinyl:benzoate coenzyme A transferase	***2.3***	***2.0***	***1.9***
Q39TW1 Polyferredoxin, putative benzoyl-CoA reductase	***2.3***	***3.9***	***4.3***
Q39ZG7 ATPase, AAA	***2.2***	***4.2***	***3.1***
Q39TP5 Cyclohexa-1,5-dienecarbonyl-CoA hydratase	***2.0***	1.3	1.0
Q39TV8 Benzoyl-CoA reductase, putative	***1.6***	1.4	1.4
Q39TW5 Benzoyl-CoA reductase electron transfer protein	***1.5***	***1.9***	***2.0***
Q39TW0 Iron-sulfur cluster-binding oxidoreductase	***1.5***	***1.5***	***1.6***
Q39TY0 Electron transfer flavoprotein-associated cytochrome b	1.1	***2.6***	**2.5**
Q39TQ2 Benzoate–coenzyme A ligase	1.1	***9.1***	***7.0***
Q39VH3 Lipoprotein release ABC transporter, membrane protein	0.1	***1.8***	***1.8***
**Butanoate metabolism**			
Q39TU7 Phosphotransbutyrylase	1.5	***0.4***	***0.4***
Q39TX4 Enoyl-CoA hydratase/isomerase	1.4	***0.4***	***0.2***
Q39UY1 Electron transfer flavoprotein, alpha subunit	0.9	***4.5***	2.6
Q39UY5 Acyl-CoA–carboxylate coenzyme A transferase, beta subunit	0.7	0.5	***0.2***
**Ethanol degradation**			
Q39WT9 Aldehyde:ferredoxin oxidoreductase, tungsten-containing	***0.2***	0.6	0.4
Q39WT8 Ethanol dehydrogenase, putative	***0.0***	0.6	0.2
**Fatty acids metabolism**			
Q39Z23 Acyl-CoA synthetase, AMP-forming	***17.6***	***52.8***	***21.9***
Q39TX1 Thiolase	***9.7***	***4.0***	***2.9***
Q39V92 Malonyl CoA-acyl carrier protein transacylase	***7.7***	***3.8***	***3.3***
Q39T45 3-hydroxyacyl-[acyl-carrier-protein] dehydratase	***6.5***	***6.8***	***2.4***
Q39V89 3-oxoacyl-[acyl-carrier-protein] synthase	***4.0***	***1.9***	***1.7***
Q39TX0 Glutaryl-CoA dehydrogenase	***3.5***	***3.6***	***2.0***
Q39TY6 Oxidoreductase, short-chain dehydrogenase/reductase family	***2.6***	***1.8***	1.2
Q39QI9 3-oxoacyl-[acyl-carrier-protein] synthase	1.8	***11.3***	***8.1***
Q39SJ7 Enoyl-[acyl-carrier-protein] reductase [NADH]	0.8	0.4	***0.1***
Q39V93 3-oxoacyl-[acyl-carrier-protein] synthase	0.8	0.6	***0.5***
**p-hydroxybenzoate degradation**			
Q39TQ0 Sigma-54-dependent transcriptional response regulator	***49.7***	***78.6***	***40.0***
Q39TQ9 4-hydroxybenzoyl-CoA reductase subunit	1.0	***2.6***	2.2
Q39TQ1 Uncharacterized protein	0.7	***1.6***	1.5
Q39TQ4 Zinc-dependent hydrolase	0.1	***2.5***	***1.4***
**Phenol degradation**			
Q39TW6 Iron-sulfur cluster-binding protein	***2.7***	***2.7***	***2.9***
Q39TU3 Phenylphosphate carboxylase, beta subunit	1.0	***3.6***	***2.7***
**Propanoate metabolism**			
Q39QL0 (R)-methylmalonyl-CoA mutase, isobutyryl-CoA mutase-like	***2.3***	1.9	1.3
Q39VB8 (R)-methylmalonyl-CoA mutase	1.1	0.4	***0.1***
Q39QK8 Methylmalonyl-CoA epimerase	***0.2***	***0.1***	***0.1***
**Pyruvate metabolism**			
Q39UG6 Citramalate synthase	***2.9***	***4.7***	***4.1***
Q39WZ8 Acetyl-CoA carboxylase	***3.0***	***6.0***	***6.6***
Q39WZ9 Acetyl-CoA carboxylase	***2.6***	2.2	***2.8***
Q39WC1 Acetyl-coenzyme A carboxylase subunit alpha	***1.8***	***1.9***	1.4
Q39SS3 Acetyl-coenzyme A carboxylase subunit beta	0.3	0.7	***0.3***
Q39R65 Succinyl:acetate coenzyme A transferase	1.0	***1.5***	1.4
Q39QU2 Phosphoenolpyruvate carboxykinase	***2.6***	***3.4***	***2.4***
Q39RG6 Pyruvate, phosphate dikinase	***2.2***	***3.9***	***3.2***
Q39S03 Pyruvate dehydrogenase complex, E1 protein, beta subunit	2.1	***9.5***	***7.4***
Q39V56 Malate oxidoreductase, NADP-dependent	1.8	***4.1***	***4.1***
Q39ZF3 Pyruvate kinase	1.4	***6.0***	***5.7***
Q39Q43 Pyruvate-flavodoxin oxidoreductase	1.4	***2.8***	***2.8***
Q39W73 2-isopropylmalate synthase	1.3	***3.0***	***3.1***
Q39XG6 Pyruvate carboxylase	1.1	***3.2***	***2.7***
Q39SB7 Phosphoenolpyruvate carboxykinase [GTP]	0.8	***1.7***	1.4
Q39RZ6 Pyruvate dehydrogenase E1 component subunit alpha	***0.6***	***0.4***	***0.3***
Q39RZ2 Dihydrolipoyl dehydrogenase	***8.4***	3.6	0.6
**TCA cycle**			
Q39S67 Citrate synthase	***6.2***	***4.5***	***2.9***
Q39VX9 2-oxoglutarate:ferredoxin oxidoreductase, ferredoxin subunit	***5.0***	3.0	0.7
Q39TX7 Succinate–CoA ligase [ADP-forming] subunit alpha	***4.8***	***3.7***	***2.0***
Q39VX8 2-oxoglutarate:ferredoxin oxidoreductase, alpha subunit	***4.7***	***3.9***	***4.3***
Q39VX7 2-oxoglutarate:ferredoxin oxidoreductase	***3.8***	***5.0***	***5.5***
Q39VX6 2-oxoglutarate:ferredoxin oxidoreductase, gamma subunit	***3.5***	1.3	0.8
Q39VY1 Isocitrate dehydrogenase, NADP-dependent	***2.8***	1.5	0.9
Q39T04 Succinate dehydrogenase/fumarate reductase	***2.5***	***3.5***	***3.4***
Q39T03 Succinate dehydrogenase/fumarate reductase	***2.3***	***10.6***	***11.1***
Q39WW6 Aconitate hydratase	1.6	0.7	***0.3***
Q39TX6 Succinate–CoA ligase [ADP-forming] subunit beta	1.4	***2.9***	***1.9***
Q39T02 Succinate dehydrogenase/fumarate reductase	0.7	***3.7***	2.7
Q39XQ3 Succinate–CoA ligase [ADP-forming] subunit beta	0.7	***0.2***	***0.2***
**Toluene degradation**			
Q39VG0 2-[hydroxy(Phenyl)methyl]-succinyl-CoA dehydrogenase subunit	***8.5***	***2.9***	2.2
Q39VF2 (R)-benzylsuccinate synthase, beta subunit	5.8	***11.0***	3.9
Q39VG2 Benzoylsuccinyl-CoA thiolase subunit	***4.9***	***13.3***	***13.0***
Q39VG1 Benzoylsuccinyl-CoA thiolase subunit	***4.6***	***3.2***	***4.5***
Q39VG4 Electron transfer flavoprotein, alpha subunit	***3.9***	1.7	1.5
Q39VG8 Succinyl:(R)-benzylsuccinate coenzyme A transferase subunit	***3.5***	***5.0***	***4.0***
Q39VG5 Electron transfer flavoprotein, beta subunit	***3.5***	1.5	1.4
Q39VG6 (E)-2-benzylidenesuccinyl-CoA hydratase	***3.5***	***3.0***	***3.0***
Q39VF5 Aromatic hydrocarbon degradation outer membrane protein	***3.0***	2.1	***2.5***
Q39VG9 Succinyl:(R)-benzylsuccinate coenzyme A transferase subunit	***2.8***	***3.2***	***3.3***
Q39VF9 2-[hydroxy(Phenyl)methyl]-succinyl-CoA dehydrogenase subunit	***2.0***	0.6	***0.5***
Q39VG7 (R)-benzylsuccinyl-CoA dehydrogenase	2.0	***4.1***	***3.9***

*For convenience averaged protein abundances of corresponding column fractions are represented relative to averaged protein abundances of high benzoate fraction and expressed as ratios. Significant values are given in bold. Significant values above a ratio of 2.5 are highlighted in orange, significant values below 0.3 are highlighted in green*.

#### Benzoate Degradation

Most of the proteins predicted for benzoate metabolism (21 proteins) were detected in all columns (Table 1). The benzoate-activating protein succinyl:benzoate coenzyme-A transferase (Gmet_2054) revealed 2-fold higher abundances at medium, low, and non-detectable benzoate concentrations relative to high benzoate concentration (Table 1). The alternative enzyme for benzoate activation, benzoate-coenzyme A ligase (Gmet_2143), had 7 to 9-fold increased abundances at low and non-detectable concentrations of benzoate relative to high concentration (Data Sheet S1). In contrast, in earlier retentostat experiments, succinyl:benzoate coenzyme A transferase (Gmet_2054) was detected at lower abundances when compared to batch, but benzoate-coenzyme A ligase (Gmet_2143) was not detected at all (Marozava et al., 2014b). Therefore, our results suggest that in the environment and in the presence of other possible benzoate-degraders *G*. *metallireducens* expresses both benzoate-activating enzymes.

#### Butanoate Degradation

Twelve proteins of the *Geobacter* genome are predicted to be involved into butyrate degradation. Six of them were detected both in column experiments and retentostats (Marozava et al., 2014b). However, butyrate-activating butyrate kinase (Gmet_2128) was not detected in the column experiments while the other butyrate-activating protein phosphotransbutyrylase (Gmet_2098) was detected and had significantly lower abundances at low and non-detectable benzoate concentrations relative to high benzoate (0.4-fold) (Table 1).

#### Ethanol Degradation

Four gene products from the *Geobacter* genome are predicted to be involved in ethanol degradation (two aldehyde dehydrogenases and two alcohol dehydrogenases). In column experiments, only ethanol dehydrogenase (Gmet_1046) and aldehyde:ferredoxin oxidoreductase (Gmet_1045) were detected (Table 1). The two enzymes were downregulated at low vs. high benzoate concentrations. This is in contrast to the earlier retentostat experiments, where all four proteins had very high abundances at low substrate concentrations and growth rates relative to batch cultures (Marozava et al., 2014b). Especially the two alternative proteins, the iron-containing alcohol dehydrogenase (Gmet_1053) and aldehyde dehydrogenase (Gmet_0789) were detected only under low growth rates in retentostats but not in batch cultures.

#### Toluene Degradation

Twelve out of 15 proteins predicted for toluene degradation by *Geobacter* were detected in all column experiments while toluene-degrading proteins were not detected in the earlier retentostat experiments (Marozava et al., 2014b). Most of the proteins for toluene degradation, including a subunit of (R)-benzylsuccinate synthase (Gmet_1538) and benzoylsuccinyl-CoA thiolase (Gmet_1528, Gmet_1529) were highly abundant at the intermediate concentration of 0.2 mM benzoate as well as at low and non-detectable benzoate concentration compared to the high concentration at the inlet (0.9 mM) (Table 1).

#### Phenol and 4-Hydroxybenzoate Degradation Pathways

Out of six predicted proteins for phenol degradation only the iron-sulfur cluster binding protein BamI (Gmet_2079) and beta subunit of phenylphosphate carboxylase were detected in column experiments and had significantly higher abundances at medium, low, and non-detectable benzoate concentrations relative to high benzoate concentrations (Data Sheet S1). In retentostat experiments four proteins from phenol catabolism were detected, including protein BamI (Gmet_2079) and phenylphosphate carboxylase, beta subunit (Gmet_2102) which was detected only at low growth rates in retentostats (Marozava et al., 2014b).

Eight proteins in the genome of *G. metallireducens* were predicted to be involved in *p*-hydroxybenzoate degradation. Four of them were detected in the columns and three in the earlier retentostat experiments (Marozava et al., 2014b and Data Sheet S1). In the column experiments, the 4-hydroxybenzoyl-CoA reductase subunit (Gmet_2136) had slightly higher abundance at low benzoate concentration relative to high benzoate concentration (2.6-fold) (Table 1). None of the three subunits of 4-hydroxybenzoyl-CoA reductase (Gmet_2134, Gmet_2135, Gmet_2136) were detected in the earlier retentostat experiments.

Neither proteins from the *p*-cresol gene cluster (Gmet_2117-2127) predicted by Peters et al. (2007) nor proteins involved in degradation of benzylalcohol and benzaldehyde (Butler et al., 2007) were detected in column or retentostat experiments (Marozava et al., 2014b and Data Sheet S1).

## Discussion

It is still unclear how microorganisms regulate the consumption of organic compounds under natural conditions. Due to the very low growth rates and substrate concentrations, chemostats have been considered as a more appropriate setup to study microbial physiology at oligotrophic conditions, in contrast to batch incubations with high substrate concentrations and maximal growth rates (Kovarova-Kovar and Egli, 1998). However, life in natural habitats includes several other important characteristics such as mixed communities and a sessile lifestyle in biofilms. Hence, we aimed at elucidating how microorganisms degrade organic substrates in sediment columns as a proxy for environmental condition in aquifers. The main question was if microorganisms in aquifers degrade one substrate at a time in a diauxic growth similar to batch cultures or if they degrade all substrates simultaneously as in chemostats.

### Previous Studies on the Physiology of *G. metallireducens*

Several studies investigated the physiology of *Geobacter* under different environmental conditions (O'Neil et al., 2008; Mouser et al., 2009; Elifantz et al., 2010). In one field experiment, relatively high concentrations of acetate (0.5–4 mM) were injected into an uranium-contaminated aquifer to study uranium reduction by *Geobacter* (Wilkins et al., 2009, 2013; Callister et al., 2010). However, no significantly upregulated proteins involved in carbon metabolism could be detected during the late phase of the experiment when acetate concentrations were lowered but were still above the detection limit (Wilkins et al., 2013).

To elucidate the physiology of *Geobacter* at low growth and low substrate concentrations, we previously compared the physiology of *G. metallireducens* in batch cultivation (excess of substrate with 1 mM benzoate and 1 mM acetate, maximal growth rate of 0.06 h^−1^) (Marozava et al., 2014b) with growth in retentostats (very low substrate concentration benzoate <20 μM, acetate <100 μM, very low growth rate of 0.002 h^−1^) (Marozava et al., 2014b). As a result, it was concluded that under low growth rates and low substrate concentrations *G. metallireducens* is adapted to simultaneously utilizing some aromatic substrates (benzoate, p-hydroxybutyrate) and fermentation products (such as ethanol, butyrate, fatty acids).

### Physiology in Columns

Proteomic analysis of *G. metallireducens* in the sediment columns experiments presented here revealed that cells were active and numerous proteins related to amino acid metabolism, ribosomal synthesis and anabolism were abundant. Even in column sections with non-detectable concentrations of benzoate, a number of proteins for translation were upregulated, indicating activity of *G. metallireducens*. This goes in line with a recent study where the toluene degrader *Pseudomonas veronii* increased amino acids synthesis when cultivated in sand, compared to slow growth rates in liquid medium (Hadadi et al., 2020).

Expression of the two benzoate-activating enzymes (succinyl:benzoate coenzyme A transferase and benzoate-coenzyme A ligase) might assist *G. metallireducens* in growth at such low substrate concentrations. The first reaction is less energetically demanding (Oberender et al., 2012) while the second one is irreversible with a high affinity for benzoate (Egland et al., 1995). The latter can be advantageous for competing at low substrate concentrations. High abundance of the benzoate-coenzyme A ligase at low concentrations in the column experiments suggests that *G. metallireducens* expresses a pathway that is competitive for lower concentrations at the expense of energy loss.

Increased production of antitoxin proteins, efflux pumps, and other cell wall-related proteins suggests that *Geobacter* undergoes a physiological adaptation to steady state conditions in the sediment (Raetz et al., 2007; Zhang and Rock, 2008). Moreover, very low numbers of detected motility proteins (1 out of 130 predicted) suggest that *G. metallireducens* has adapted to a sessile life style in the sediment.

### De-repression of Catabolic Pathways in Columns

**Figure 4:**
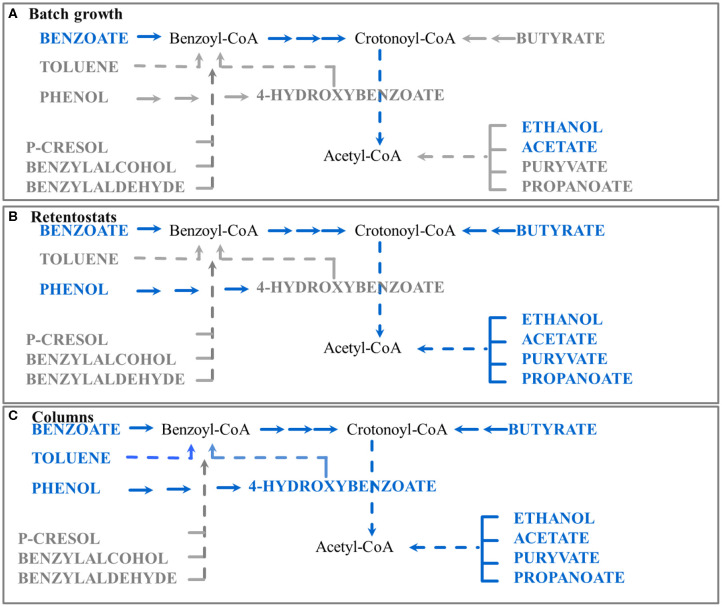
Schematic representation of the proposed regulation of catabolic pathways by *G.metallireducens* under different conditions studied to date (batch, retentostat, and sediment column). Blue color represents substrates which are readily used by *G. metallireducens*, gray color represents substrates where degradation might need specific induction and no proteins were detected for their degradation. Proteins of the corresponding pathways were detected in **(A)** batch experiments with benzoate as a carbon source (Marozava et al., 2014a); **(B)** retentostats with acetate plus benzoate as carbon sources (Marozava et al., 2014b); **(C)** columns with benzoate as a carbon source (this study).

Even in the absence of toluene in the inflow of the columns, *G. metallireducens* significantly upregulated the complete toluene degradation pathway in column sections of benzoate concentrations below 0.2 mM. Moreover, key enzymes for phenol and 4-hydroxybenzoate degradation such as iron-sulfur cluster binding protein BamI and 4-hydroxybenzoyl-CoA reductase were also abundant (Data Sheet S1). Hence, we suggest that regulation of toluene and 4-hydroxybenzoate degradation pathways is independent from the presence of the corresponding substrates toluene and 4-hydroxybenzoate (Figure 4). Surprisingly, these pathways were not detected at low growth rates in retentostats (Marozava et al., 2014b).

*Geobacter* species have a complex system of regulating their metabolism with many sigma factors and over 100 translation regulators (Lovley et al., 2011). However, molecular mechanisms regulating the carbon metabolism are still not well-understood. Some sigma factors are involved in adaptation to changing environments in *G. metallireducens* (Reguera and Kashefi, 2019) but it is unknown which global regulators are responsible for derepressing or upregulating catabolic pathways during carbon limitation. The RpoS sigma factor related to viability of *Geobacter* in the stationary phase, is involved in synthesis of pentaphosphate (pppGpp) and triphosphate (ppGp) alarmones during nutrient limitation, and causes slowed growth (Reguera and Kashefi, 2019). To our knowledge, there is no indication if RpoS is involved in the release of catabolite repression at low substrates concentrations.

Some regulators are known to be related to the control of aromatic degradation pathways. For example, the BgeR transcriptional regulator represses expression of the benzoate degradation pathway of *Geobacter bemidjiensis* during growth with acetate, and this repression is relieved again in the presence of benzoate (Ueki, 2011). BgeR is also expected to play an important role in the expression of other aromatic degradation pathways but it is still unknown to which extent. In our sediment columns, a product of the other transcriptional regulator gene *bamW* (Gmet_2145) (Juarez et al., 2010) had 40 to 70-fold higher abundances at medium, low, and non-detectable benzoate concentrations relative to high benzoate concentration at the inflow. This regulator protein also showed increased expression in response to the aromatic substrates p-cresol, phenol, or benzoate in batch cultures of *Geobacter* (Peters et al., 2007; Schleinitz et al., 2009). However, the product of the *bamW* gene was not detected at very low benzoate concentrations in our previous retentostat study with acetate plus benzoate (Marozava et al., 2014b). Hence, it might be that the toluene degradation pathway was not detected in retentostats because the presence of acetate had some carbon catabolite repression effect. Such repression was eliminated completely when benzoate (and not acetate) was used as a sole carbon source in the column experiments.

In general, carbon catabolite repression is probably relieved when concentrations of the preferred substrate drop below certain thresholds but the specific molecular mechanism needs further investigation.

Only few studies exist on the regulation of the peripheral pathway of toluene degradation in anaerobic bacteria. Recently, it has been shown that the nitrate-reducing bacterium *Azoarcus* strain CIB repressed expression of benzylsuccinate synthase in the presence of benzoate (Blázquez et al., 2018). However, our batch experiments did not suggest any catabolite repression of toluene in the presence of benzoate since these substrates were consumed simultaneously by *G. metallireducens* in batch (Marozava et al., 2014a). Moreover, basal expression of almost all toluene degradation proteins was observed when *G. metallireducens* was cultivated in batch with the substrates acetate, benzoate, ethanol, or butyrate (Marozava et al., 2014a). None of these substrates induced a similar strong expression of the toluene degradation pathway as toluene only (Marozava et al., 2014a) or as cultivation in the columns at measured benzoate concentrations below 0.2 mM. Moreover, mRNAs of benzylsuccinate synthase alpha and beta subunits were found to be two times more abundant with benzoate relative to acetate when *G. metallireducens* was cultivated in chemostats (Butler et al., 2007). However, abundances of benzylsuccinate synthase with toluene as substrate relative to acetate were found to be up to 2,000-fold higher in batch cultures (Marozava et al., 2014a). This suggests that benzoate promotes expression of the toluene degradation pathway to some extent but toluene itself is a much stronger inducer. In the denitrifying bacterium *Thauera aromatica* toluene-degrading proteins have been shown to be significantly induced not only by toluene but also in the presence of benzylalcohol or benzylaldehyde (Biegert and Fuchs, 1995). Whether this is a case for *G. metallireducens* we do not know.

The metagenomes of the other taxa detected in our columns showed some aerobic benzoate degradation pathways (Data Sheet S1). However, since the columns were run under anoxic conditions these pathways were probably not relevant. The only molecular oxygen in the system might have been produced from nitrate reduction via nitric oxide dismutases, since it has been shown recently that these enzymes are abundant in different environments but have been strongly overlooked (Zhu et al., 2020). In other mixed communities where benzene was degraded under nitrate-reducing conditions, both anaerobic and aerobic pathways for benzene degradation were found and the role of nitric oxide dismutase was highlighted (Atashgahi et al., 2018). Based on the present data, however, we were not able to assess whether bacteria from a microbial community expressed functionally active nitrite oxide dismutases in the columns. Nevertheless, *Geobacter* can be a good co-existing partner for other bacteria and can even stimulate methane production in microbial electrolysis cells (Yin et al., 2016).

We conclude that low substrate concentrations in combination with low growth rates and probably some other factors present in the environment such as mixed communities and a sessile lifestyle, prepare cells of *G. metallireducens* for toluene degradation.

### Model for Regulation of Catabolic Pathways of *G. metallireducens* in the Environment

Based on our previous data and the column experiments presented here, we suggest an updated model for regulation of catabolic pathways of *G. metallireducens* in the environment as a response to carbon concentration and at sessile life style. At very low substrate concentrations below 20 μM in retentostats, *G. metallireducens* co-expresses the pathways for utilizing the fermentation products acetate, pyruvate, butyrate, ethanol, and propionate, as well as the aromatic compounds benzoate and phenol (Figure 4B). At benzoate concentrations below 0.2 mM and a sessile life style in sediment columns, *Geobacter* furthermore expresses pathways for utilizing toluene and 4-hydroxybenzoate (Figure 4C). Expression of p-cresol, benzyl alcohol, and benzaldehyde degradation pathways still requires specific induction and presence of the respective substrates in the environment (Figure 4). Hence in the environment, the physiology of *G. metallireducens* at benzoate concentrations below 0.2 mM is more similar to its physiology in retentostats (at concentrations below 20 μM) and does not reflect diauxic growth in batch cultures (at initial concentrations of 1 mM) (Figure 4). A concentration of 0.2 mM benzoate might seem high compared to the 10-fold lower concentration in retentostats. However, in the sediment, the actual benzoate concentration around the sessile *G. metallireducens* cells or in biofilms might be lower due to e.g., diffusion limitation or benzoate consumption of the competing degraders from the mixed community. Similar to *Aromatoleum aromaticum* EbN1, which is ready to consume even nanomolar concentrations of alkylphenols (Vagts et al., 2020), *G. metallireducens* prefers to scavenge the environment for alternative substrates as a way to survive carbon limitations.

Thus, we propose that organisms such as *G. metallireducens* are specialized on a preferred range of substrates which they express at low substrate concentrations even if the respective substrates are absent. Under such environmental conditions, they express their own specific standard repertoire of degradation pathways, which will be different for every organism. This tendency is even more pronounced in sediment columns reflecting rather close to natural conditions compared to chemostats or even retentostats. Our findings also indicate a preference of *G. metallireducens* to degrade aromatic hydrocarbons in the presence of naturally occurring fermentation products. Although such a strategy might seem to be energetically unfavorable, it has been suggested that in such way organisms adapt to dynamic conditions in the environment and might be in line with optimized growth (De Groot et al., 2019).

## Data Availability Statement

The datasets presented in this study can be found in online repositories. The names of the repository/repositories and accession number(s) can be found in the article/Supplementary Material.

## Author Contributions

SM and RM conceived the idea and wrote the manuscript. SM performed the column experiments and analyzed the data. HM conducted metagenomics. JM-P performed metaproteomics. All authors contributed to the article and approved the submitted version.

## Conflict of Interest

The authors declare that the research was conducted in the absence of any commercial or financial relationships that could be construed as a potential conflict of interest.

## Supplementary Material

Data Sheet S1 All proteins and their abundances detected in all column fractions.
