# Supplementary material for: Adaptation of Carbon Source Utilization Patterns of Geobacter metallireducens During Sessile Growth
Source: Front Microbiol. 2020 Jun 23;11:1271. doi: 10.3389/fmicb.2020.01271 (PMC7324539; doi:10.3389/fmicb.2020.01271)
Supplement: Data Sheet S1 — All proteins and their abundances detected in all column fractions. [file Data_Sheet_2.docx]

**Supplementary information**

**
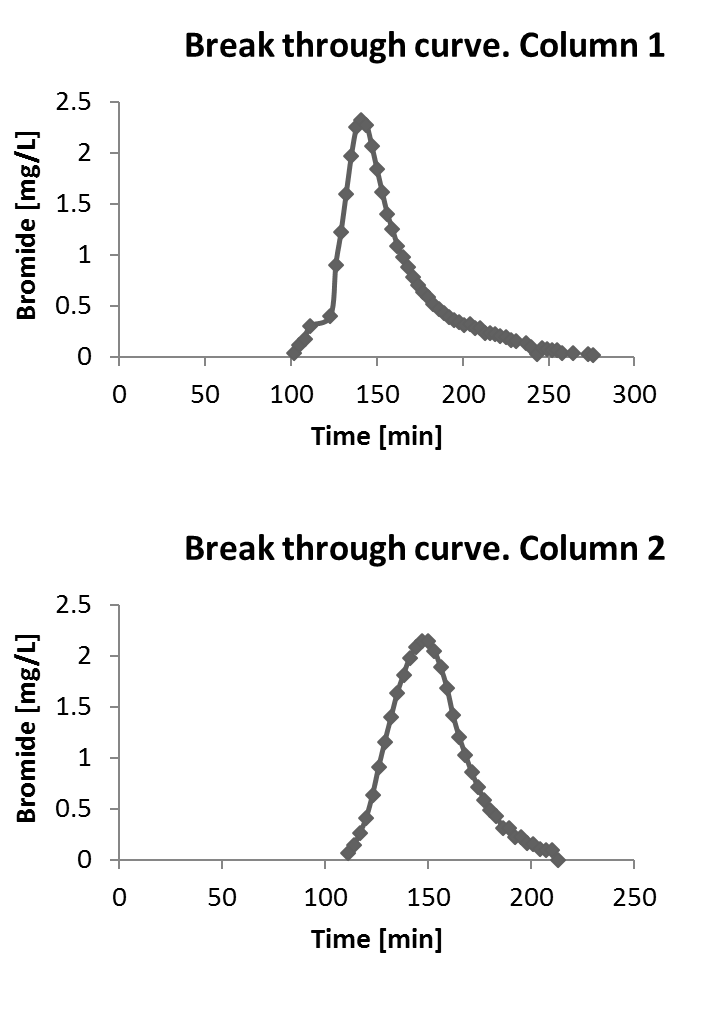
**

**Fig. S1.** Breakthrough curve of tracer bromide and benzoate in uninoculated Columns 1 and 2.


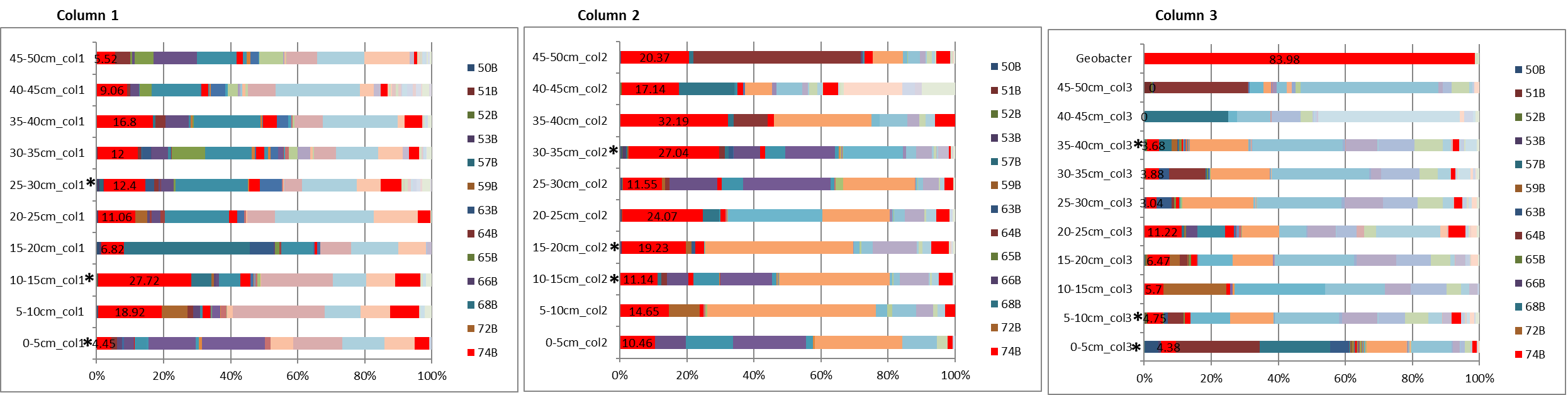


**A**

**
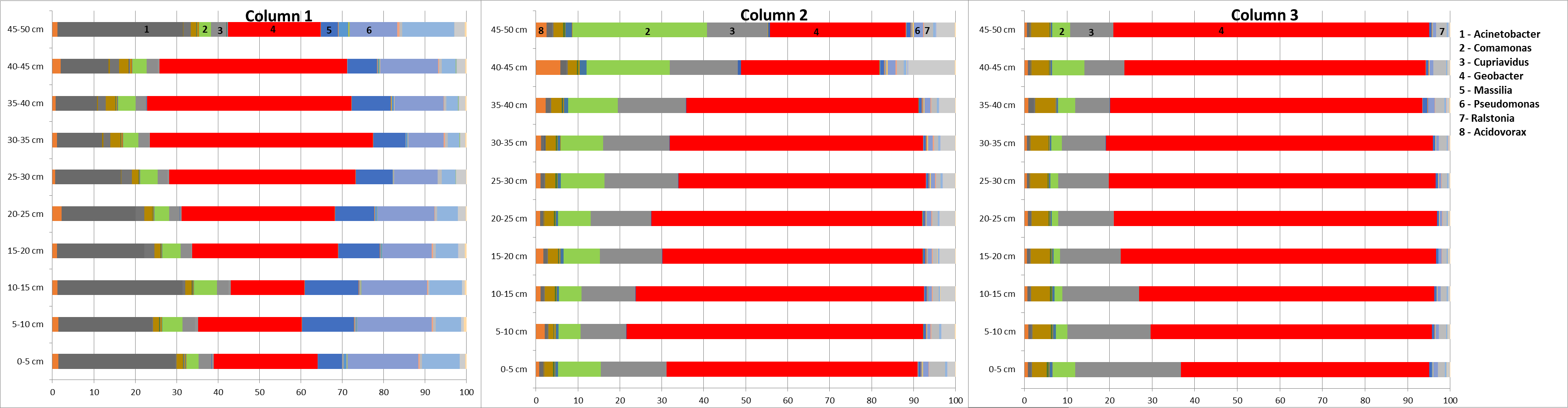
B**

**Fig. S2 A.** Bacterial 16S rRNA gene T-RFLP fingerprints of bacterial communities in column fractions and of a pure culture of *G. metallireducens* (indicated in column 3). Abundances in red colour represent 16S rRNA of *G. metallireducens*. Fractions selected for metagenomic analysis are labelled with a star (*).

**Fig. S2 B.** Metaproteomic fingerprint of communities in the sediment across height of the columns expressed as relative protein abundance, measured as a percentage of normalized protein abundances of a given organism from all the proteins detected. Fingerprints of the most abundant Genus are indicated with numbers at 45-50 cm depth with their names indicated in a legend. Fingerprints of *G. metallireducens* are indicated in red.


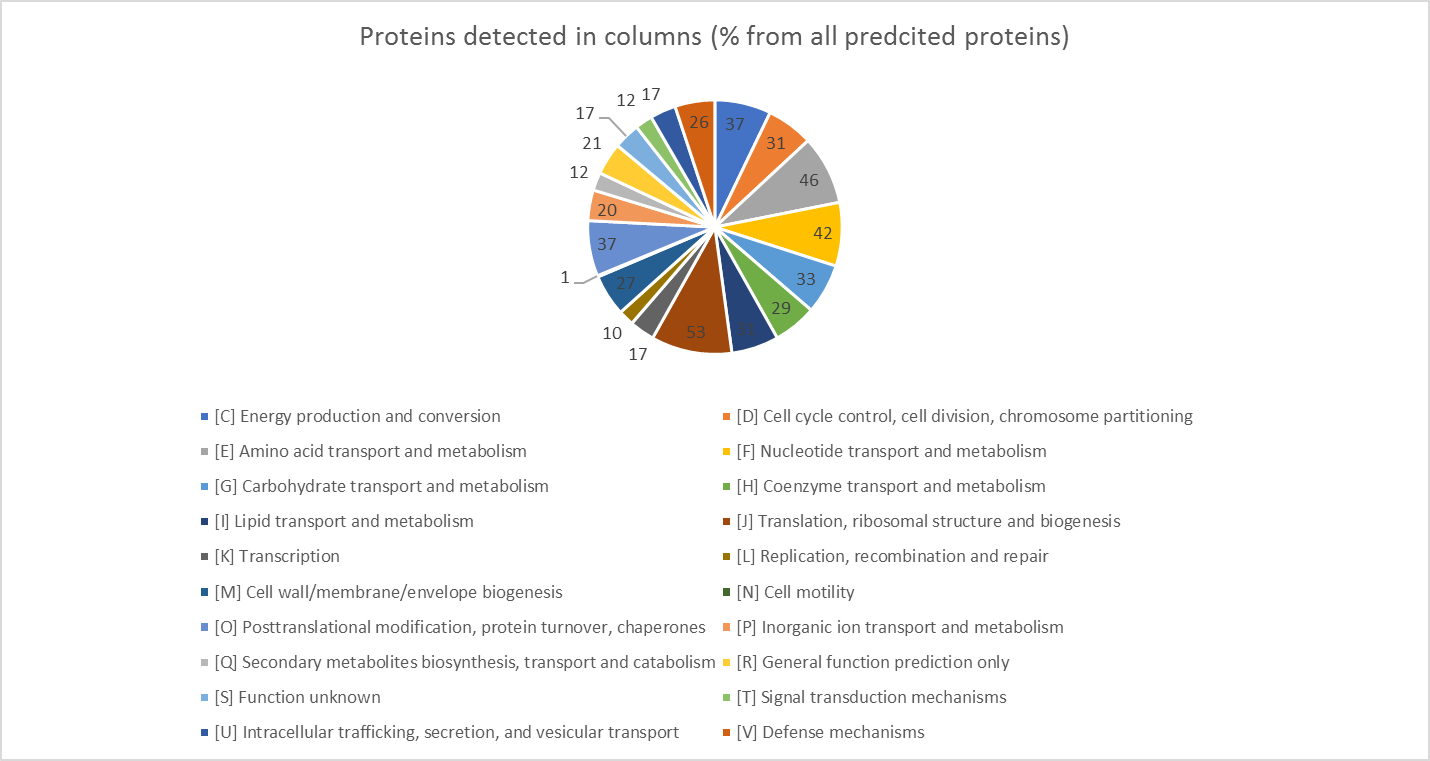


Fig. S3. Distribution of detected proteins in columns according to the COG classification. The figure depicts the percentage of detected proteins of all predicted in the *Geobacter* genome.

**
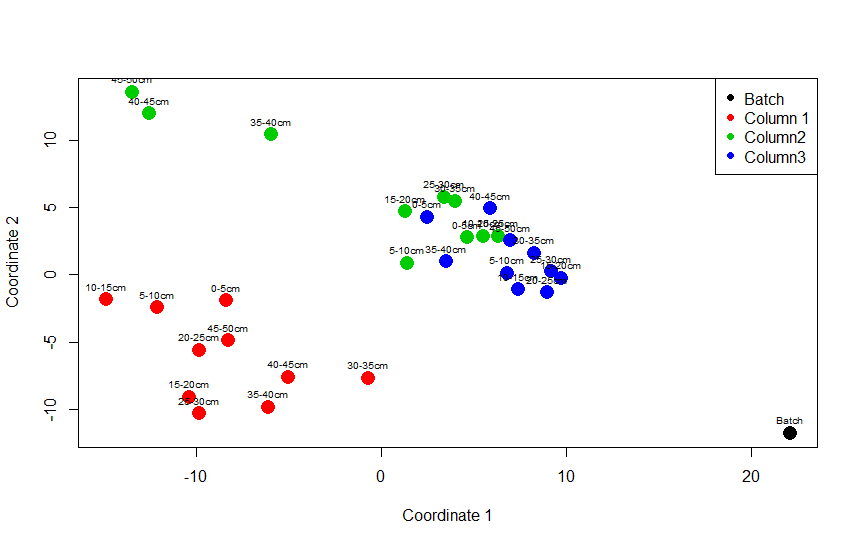
**

**Fig. S4.** Kruskal's Non-metric Multidimensional Scaling (nMDS) of protein abundances of all proteins detected in column experiments and in batch. Four dimensions were used for nMDS and 3.4 % of stress has been computed.


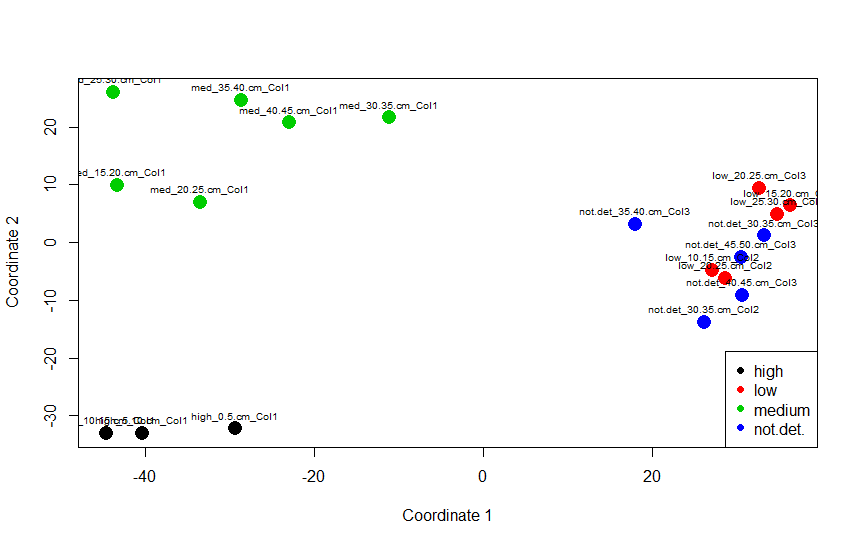


**Fig. S5.** Kruskal's Non-metric Multidimensional Scaling (nMDS) of protein abundances of selected depths in column experiments. Four dimensions were used for nMDS and 3.4 % of stress has been computed.


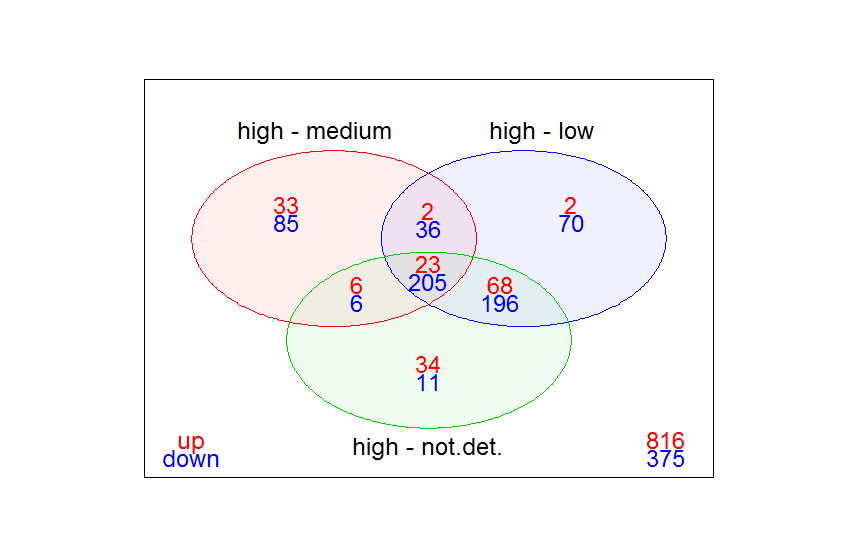


**Fig. S6.** Venn diagram depicting numbers of differentially expressed proteins in pair wise comparisons. Each circle represents the number of proteins which appeared to be significantly different in the corresponding pair wise comparisons: high benzoate concentration vs medium concentration; high benzoate concentration vs low benzoate, high benzoate concentrations vs zone with no benzoate detected. Red colour in the circle (upper value) indicates the number of up-regulated proteins while blue colour in the circle (lower value) indicates the number of down-regulated proteins.


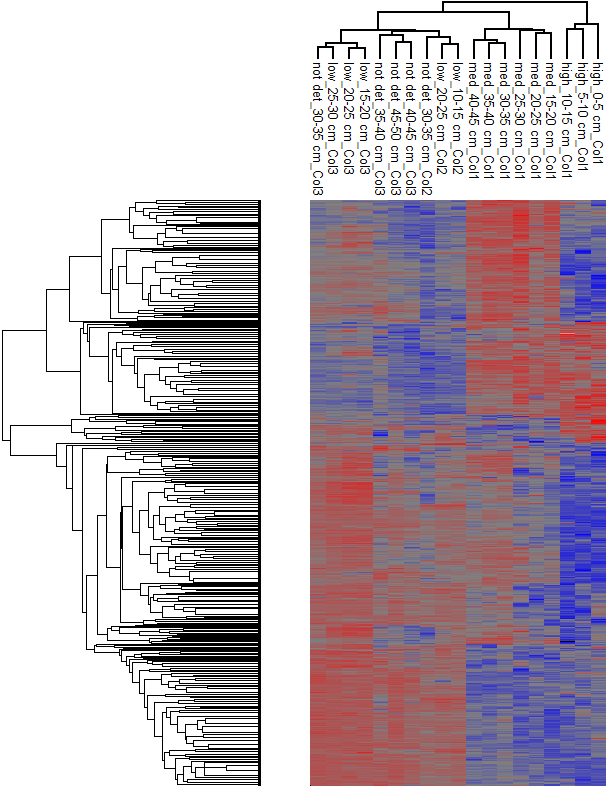


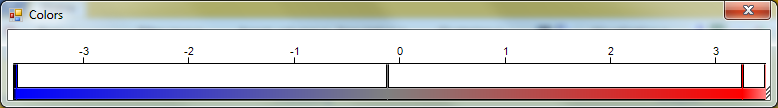


z-scores (scores of standard normal distribution) of protein abundances

**Fig. S7** Heatmap clustering of significant protein abundances detected at least in one of the pairwise comparisons.
